# Rapid generation of an anthrax immunotherapeutic from goats using a novel non-toxic muramyl dipeptide adjuvant

**DOI:** 10.1186/1476-8518-5-11

**Published:** 2007-10-22

**Authors:** Cassandra D Kelly, Chris O'Loughlin, Frank B Gelder, Johnny W Peterson, Laurie E Sower, Nick M Cirino

**Affiliations:** 1 Wadsworth Center, New York State Department of Health, Biodefense Laboratory, Albany, NY, USA; 2 SUNY at Albany, School of Public Health, Department of Biomedical Sciences, Albany, NY, USA; 3 Virionyx Corporation Ltd, Auckland, NZ, USA; 4 The University of Texas Medical Branch, Galveston, TX, USA

## Abstract

**Background:**

There is a clear need for vaccines and therapeutics for potential biological weapons of mass destruction and emerging diseases. Anthrax, caused by the bacterium *Bacillus anthracis*, has been used as both a biological warfare agent and bioterrorist weapon previously. Although antibiotic therapy is effective in the early stages of anthrax infection, it does not have any effect once exposed individuals become symptomatic due to *B. anthraci*s exotoxin accumulation. The bipartite exotoxins are the major contributing factors to the morbidity and mortality observed in acute anthrax infections.

**Methods:**

Using recombinant *B. anthracis *protective antigen (PA83), covalently coupled to a novel non-toxic muramyl dipeptide (NT-MDP) derivative we hyper-immunized goats three times over the course of 14 weeks. Goats were plasmapheresed and the IgG fraction (not affinity purified) and F(ab')_2 _derivatives were characterized *in vitro *and *in vivo *for protection against lethal toxin mediated intoxication.

**Results:**

Anti-PA83 IgG conferred 100% protection at 7.5 μg in a cell toxin neutralization assay. Mice exposed to 5 LD_50 _of *Bacillus anthracis *Ames spores by intranares inoculation demonstrated 60% survival 14 d post-infection when administered a single bolus dose (32 mg/kg body weight) of anti-PA83 IgG at 24 h post spore challenge. Anti-PA83 F(ab')_2 _fragments retained similar neutralization and protection levels both *in vitro *and *in vivo*.

**Conclusion:**

The protection afforded by these GMP-grade caprine immunotherapeutics post-exposure in the pilot murine model suggests they could be used effectively to treat post-exposure, symptomatic human anthrax patients following a bioterrorism event. These results also indicate that recombinant PA83 coupled to NT-MDP is a potent inducer of neutralizing antibodies and suggest it would be a promising vaccine candidate for anthrax. The ease of production, ease of covalent attachment, and immunostimulatory activity of the NT-MDP indicate it would be a superior adjuvant to alum or other traditional adjuvants in vaccine formulations.

## Background

*Bacillus anthracis*, the causative agent of anthrax, has been the focus of much research and attention following the release of spores through the US mail system in 2001. 22 cases of infection resulted in 5 deaths, causing much concern regarding treatment, therapeutics and vaccine efficacy. Recently, the CDC discontinued the administration of the current anthrax vaccine (Anthrax Vaccine Adsorbed -AVA) due to adverse side effects observed in a large percentage of volunteers. This revocation of available vaccine has left healthcare workers, laboratory personnel and first responders with only limited means of protection following potential exposures to anthrax spores.

In humans, the anthracis bacilli can cause three types of infections: cutaneous via abrasions in the skin, gastrointestinal through ingestion of spores in contaminated meat and inhalation when spores less than 5 uM um are deposited into the lungs [1]. The mortality rates vary between each form of the disease with cutaneous anthrax presenting as a self-limiting and treatable infection with only a 20% case fatality rate. When left untreated gastrointestinal infections can progress rapidly and have over 80% case fatality rates. Inhalation anthrax infections are rare but have a high case fatality rate (over 75%) even with antibiotic treatment.

Treatment options for patients presenting with symptoms of inhalational anthrax infections are limited and are generally ineffective at reducing mortality. Although antibiotic therapy is effective in the early stages of infection, it does not have any effect on the bipartite exotoxins, which are the major contributing factors to the mortality observed in acute anthrax infections [1]. The current lack of an approved, available vaccine puts laboratory workers, military personnel and first responders at an increased risk of inhalational anthrax should another terrorist event, similar to the anthrax mailings in 2001, occur. Clearly there is a need for an effective vaccine as well as a well-tolerated, economical, post-exposure therapeutic for the treatment of human anthrax infections.

Passive immunotherapy is a non-chemical therapeutic providing immediate immunity to infectious agents and toxins. This treatment option has been shown to be effective against many diseases including anthrax [2-6] and other biothreat agents [7,8]. Several approaches have been used previously for the production of immunotherapeutics specific for *B. anthracis *although they all have significant drawbacks. The pooling of immune serum from previously vaccinated volunteers yields highly protective anti-sera in very small quantities, limiting its use as a source of therapeutics for the Strategic National Stockpile or as a commercially available product. Monoclonal antibodies are highly specific, limiting their application to a single antigenic target and have a high cost associated with their development further limiting their feasibility for mass production and stockpiling. In the past animal vaccination has successfully been used to generate immunotherapeutic antiserum specific for infectious and toxic agents including snake venom, botulism toxin and Ebola virus [9-12] but limitations in quantity and safety have prevented their widespread use in the development of human therapeutics. Horses can provide large amounts of antiserum but are costly to maintain. Mice, rabbits and guinea pigs are inexpensive to maintain but yield limited volumes of anti-sera. Goats provide a renewable source of plasma and serum; however they have not been traditionally used in the generation of passive immunotherapeutics. We have plasmapheresed hyper immunized goats to successfully produce liters of GMP-grade antisera following a short immunization schedule (3 immunizations over 14 weeks), with minimal cost.

*Bacillus anthracis *produces two separate exotoxins, edema toxin (EdTx) and lethal toxin (LeTx). The two exotoxins utilize a common cell binding component termed protective antigen (PA83, 83 kDa) which binds to the ubiquitous anthrax toxin receptor (ATR) found on most cell surfaces. Once PA83 is bound to the host cell surface, a furin-like protease cleaves the full-length, inactive protein into the active form, PA63 (63 kDa), thereby exposing the binding sites for the catalytic components of the exotoxins (edema factor, EF or lethal factor, LF). A heptamer composed of PA63 + three LF/EF moieties [13,14] forms on the cell surface and is internalized via receptor mediated endocytosis. The subsequent decrease in pH within the endosome causes conformational changes in PA63, so that it inserts into the endosomal membrane, forming a protease-stable pore; formation of this pore allows EF and LF to enter the cell and exert their toxic effects [15]. LeTx is formed when PA63 is combined with LF, and is responsible for the most severe intoxicative effects of anthrax infection. EF is an adenylate cyclase capable of causing severe disregulation of cellular cAMP levels [16]. LF has been shown to be a zinc-dependant metalloprotease with specificity for mitogen-activated protein kinase kinases (MAPKKs) capable of disrupting several cell signaling cascades; however, its specific mode of action is still unclear [17,18]. Disruption of the binding of PA to ATR or LF would disrupt internalization of functional LeTx and would thereby prevent toxin-mediated death of the host following rapid multiplication of the bacilli.

Here we immunized goats with recombinant PA83, coupled to a novel non-toxic muramyl dipeptide derivative (NT-MDP) capable of inducing both innate and humoral immunity and does not induce clotting even when administered at high concentrations. The resulting polyclonal anti-sera conferred protection against *in vitro *and *in vivo *intoxication with the anthrax lethal toxin (LeTx) and *in vivo *intranasal challenge with virulent *B. anthracis *spores. Recently, we have shown that the passive transfer of goat-derived anti-HIV antibodies to failing therapy AIDS patients has been well tolerate, safe and effective [19-21].

In order to circumvent any hypersensitivity reactions associated with goat IgG, we have explored the use of F(ab')_2 _antibodies lacking the Fc region of the IgG molecule. The Fc region of the IgG is involved in the activation of complement, and patients with a pre-developed sensitivity to goat proteins may be at a higher risk of developing fatal allergic reactions following the administration of a goat-based antibody therapy. Removal of the Fc region allows for the retention of the dimeric antigen binding sites while increasing the safety of the immunotherapeutic without a significant loss in neutralizing capabilities.

Our data suggests that the administration of anti-PA83 goat IgG or F(ab')_2 _would provide an efficacious and well-tolerated passive immunotherapy for post-exposure treatment of acute human anthrax infections. Most notable is the rapidity with which the anti-sera were produced in goats and the volume of anti-sera generated from a single plasmapheresis. In addition, this data serves a proof of concept that a rapid, inexpensive, GMP-grade immunotherapeutic can be produced in a short enough timeframe for an emerging disease event like SARS-CoV.

## Methods

### Recombinant anthrax toxin proteins

High-purity, histidine-tagged rLF and rPA83 were supplied by the Northeast Biodefense Center Protein Expression Core. Functional lethal toxin (LeTx) was formed by the combination of purified rLF and rPA83 at a 1:1 (w/w) ratio diluted in sterile PBS.

### Caprine antisera

Purified rPA83 was supplied to Virionyx Corporation Ltd (Auckland, NZ) for caprine immunizations as follows. A novel muramyl dipeptide adjuvant (NT-MDP) was oxidized with sodium meta periodate (0.5 M) for 1 h and excess sodium meta periodate was removed by centrifugation followed by a water wash. 1 mg of rPA83 in sodium carbonate buffer (0.1 M, pH 9.5) was added to 10 mg of activated NT-MDP and incubated overnight at room temperature. The resulting Schiff's base was reduced by the addition of ascorbic acid to achieve a pH of 7.0. Three goats were immunized with 100 μg rPA83-NT-MDP conjugates emulsified in Freund's complete adjuvant and were subsequently boosted three additional times with immunogen in Freund's incomplete adjuvant over a 13-week period. Hyper-immune plasma was collected from each animal two weeks following the last immunization. Plasma was pooled and IgG was purified using a standard octanoic acid precipitation technique. Purified anti-PA83 IgG was supplied at a concentration of 15 mg/ml.

### Generation of F(ab')_2 _antibody fragments

F(ab')_2 _fragments were generated by pepsin digestion (100 U/mg IgG) at pH 3.5 in 0.1 M glycine buffer for 24 h. Reactivity was demonstrated using an Ouchterlony gel diffusion assay and demonstrated reactivity at 1 mg/ml against rabbit anti-goat IgG (data not shown). Purity and extent of digestion was determined by SDS-PAGE analysis (data not shown).

### Anti-sera titer determination

ELISAs were performed in microtiter plates coated with rPA83 (10 nM) in 10 mM carbonate/bicarbonate buffer (pH 8.5) with a final coating volume of 50 μl. Plates were coated for 1 h then washed in water and blocked with 5% non-fat milk powder. Antibody titers were measured by reacting (2 h) serially diluted anti-PA83 IgG with the rPA83-coated microtiter wells. The wells were then washed with water and reacted (2 h) with horseradish peroxidase-labeled rabbit anti-goat IgG. Following one water wash, the wells were reacted (30 min) with the substrate, orthophenylenediamine. The reaction was stopped by the addition of sulfuric acid and absorbance was measured at 492 nm. Anti-PA83 IgG titers were measured and expressed as the reciprocal of the antibody dilution which produced an absorbance value equal to 50% maximum absorbance.

### Cell lines and media

Murine macrophage-like cells, J774A.1, were obtained from the American Type Cell Culture Collection (ATCC TIB-67). Cells were cultured in complete medium: Dulbecco's Modified Eagle Medium (DMEM) supplemented with 10% fetal bovine serum, Glutamax, and penicillin/streptomycin at 37°C with 5% CO_2_.

### *In vitro *cytotoxicity and protection assays

Macrophage-like cells were harvested by gentle scraping (no trypsin) and were seeded in 96-well plates at a density of 6 × 10^4 ^cells/well in 100 μl of complete medium. Cells were incubated for 18–24 h or until > 90% confluency had been achieved. Medium was removed, and cells were washed once in sterile PBS before addition of toxin or anti-sera. For toxicity assays, 100 μl of LeTx was added to the cells at final concentrations of 1000 ng, 100 ng, 10 ng and 0.1 ng (data not shown). For protection assays, 50 ng of LeTx (2 TCEC_50_) was combined with varying dilutions of anti-PA83 IgG or F(ab')_2 _and incubated at 37°C, while shaking for 1 h prior to the addition of 100 μl per well. Cells with LeTx alone or in combination with anti-sera were incubated at 37°C and 5% CO_2 _for 4 h. Cell viability was determined using Sigma's Cell Growth Determination Kit, an MTT-based assay. Briefly, 10 μl of MTT dye was added to cells and incubated for 15 h at 37°C and 5% CO_2_. 100 μl of solubilization solution was added to each well after removal of media, and cell viability was measured at 570 nm. Percent relative cell viability was calculated as the ratio between LeTx-treated cells (LeTx) and untreated control cells (100 μl PBS). Percent protection conferred by caprine anti-PA83 IgG or F(ab')_2 _was measured as follows:

(1-((PBS - α PA83 IgG)/(PBS - 50 ng LeTx))) × 100.

### *In vivo *protection assays

#### Lethal toxin challenge

Female Balb/c mice (average weight 17.5 g) were injected with 100 μg LeTx in 200 μl saline via intraperitoneal injection (5 per group). Five minutes following toxin injection mice were injected on the opposite side with 8 mg/kg anti-PA83 IgG or F(ab')_2 _in 200 μl saline. Control mice (3 in group) received LeTx followed by saline injections. Mice were observed for signs of illness and distress for 11 days at which point all surviving mice were sacrificed.

#### Virulent *B. anthracis *spore intranasal challenge

Female Swiss Webster mice (average weight 25.2 g) were infected with approximately 5 × 10^4 ^*B. anthracis *Ames spores (5 LD_50_) by 20 μl installations in each nares. Groups of 10 mice received saline at 1 hour post-infection or anti-PA83 IgG at 24 h post-infection (32 mg/kg) by intraperitoneal injection. Mice were monitored twice daily for 14 d for signs of illness and death. To evaluate synergistic effects of antibiotic treatment post-exposure, low-dose Ciprofloxacin was administered twice daily at 0.9 mg/day via intraperitoneal injection for the first six days post spore challenge.

#### Statistical Analysis of *in vivo *results

Statistical analysis (logrank test) of the *in vivo *survival data was performed using GraphPad Prism (version 4.03), GraphPad Software, San Diego, CA.

## Results and Discussion

### Anthrax lethal toxin activity

Purified rLF (90 kDa) and rPA83 (83 kDa) showed high product purity, with no significant breakdown products by SDS PAGE, trypsin digestion and mass spectroscopy (> 95% purity for both, data not shown). *In vitro *bioactivity of LeTx was confirmed by treating J774A.1 murine macrophage-like cells with varying doses of LeTx (10 – 0.001 ng/μl), and cell viability determined via toxin neutralization assay. Cell viability experiments established a TCEC_50 _of 25 ng LeTx (equivalent to 2.85 nM, data not shown). This dose of LeTx is within the range of previously reported TCEC_50_s [22-25]. Based on this data, all subsequent *in vitro *protection assays were performed at 2× TCEC_50 _equivalent to a total of 50 ng LeTx per well.

### Generation and evaluation of anti-PA83 caprine immunoglobulin

**Figure 1:**
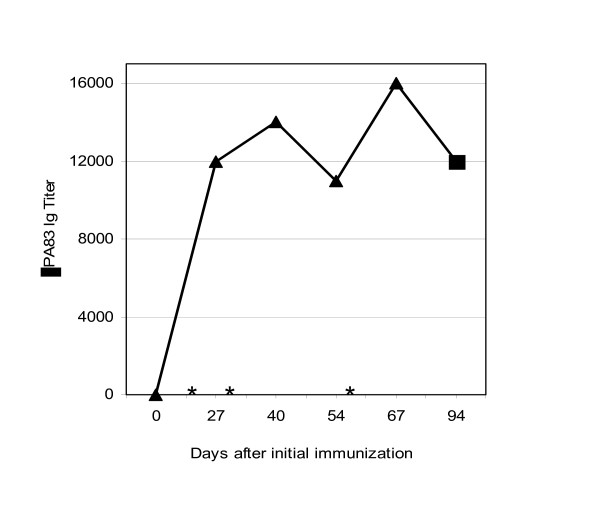
Goat anti-PA83 IgG titer. Serially diluted goat anti-PA83 IgG reacted with 10 nM rPA83 in a microplate ELISA. Titer calculated as the reciprocal of the dilution producing 50% maximum absorbance. Day 0 is 1^st ^immunization with PA83-NT-MDP, asterisks indicate timings of 2^nd ^(day 14), 3^rd ^(day 28) and 4^th ^(day 56) booster immunizations. Purified anti-PA83 IgG was obtained from plasmapheresed goats on day 94 (time point designated by a square).

One goal of this study was to produce large volumes of high titer, hyper-immune goat sera in a short period of time. Goats were immunized four times (days 0, 14, 28, 56) over a period of 56 days and subsequently plasmapheresed (day 94). Total IgG was purified from plasma and rPA83 specificity was confirmed by Western blot and ELISA (data not shown), validating the efficacy of the immunogen/adjuvant, immunization schedule, and IgG purification methods established previously with the anti-HIV immunotherapeutic [19-21]. Specific rPA83 titers were obtained from immunized goats on days 0, 27, 40, 54, 67, and 94. Antibody titers were measured by ELISA by reacting serially diluted anti-PA83 IgG with 10 nM rPA83. Anti-PA83 IgG demonstrated significant titer (> 10,000, calculated as the reciprocal of the dilution producing 50% maximum absorbance) within 2 weeks (27 d post-immunization), and reached a maximum of ~16,000 after the fourth immunization (Fig. 1). High titer polyclonal antisera could be generated in as little as 42 days thus establishing that rapid production of target-specific caprine immunotherapeutics using the novel NT-MDP adjuvant is achievable.

### Anti-PA83 IgG and F(ab')_2 _protect cells against LeTx-induced cytotoxicity

The protective efficacy of the anti-PA83 IgG and the F(ab')_2 _derivative was evaluated in the J774A.1 LeTx *in vitro *model. Cells were exposed to 0.5 ng/μl of LeTx and dilutions of anti-PA83 IgG or F(ab')_2_. MTT-based cell viability assays were used to determine percent protection as described in Materials and Methods. Control included untreated cells (i.e., PBS substituted for LeTx), cells treated with IgG alone (7.5 μg α PA83 Ig with no LeTx), or cells treated with 0.5 ng/μl LeTx alone (LeTx). LeTx treated cells demonstrated a statistically significant decrease in cell viability (p < 0.001) as compared to the untreated PBS control cells, while standard concentrations of anti-PA83 IgG (7.5 μg) had no effect on cell viability (data not shown). The use of higher concentrations of anti-PA83 IgG (up to 250 μg) produced no significant differences in cell viability (data not shown). These results confirm that caprine IgG exhibits no inherent cytotoxic effects *in vitro *and does not interfere with the observed cytotoxicity of the recombinant LeTx.

**Figure 2:**
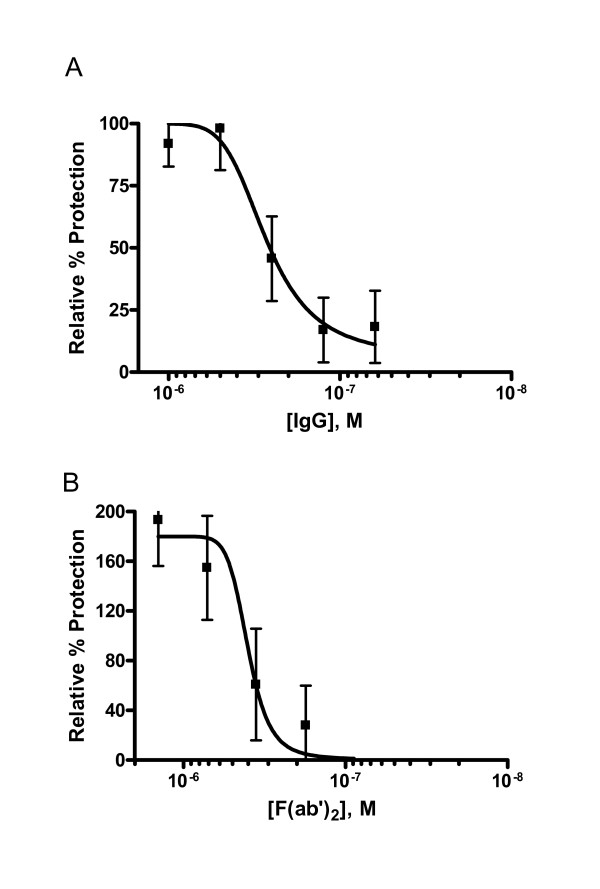
*In vitro *protection against LeTx cytotoxicity. J774A.1 cells were treated with 50 ng (~2.9 nM) LeTx and varying concentrations of goat anti-sera. Cell viability determined by an MTT-based assay. **A**. Anti-PA83 IgG. Data shown are the average ± SEM of five assays each with four replicates. EC_50 _is 2.57 × 10^-7 ^M. **B**. Anti-PA83 F(ab')_2 _fragment. Data shown are the average ± SEM of three assays each with four replicates. EC_50 _is 4.0 × 10^-7 ^M, comparable to full length IgG. Curves and EC_50 _were generated using GraphPad Prism^® ^V4.03.

Cells treated with varying concentrations of anti-PA83 IgG exhibited protection from LeTx cytotoxicity in a dose-dependant manner (Fig. 2A). Cells were exposed (five separate assays each with four replicates) to varying doses of anti-PA83 IgG and 50 ng LeTx for 4 h. 7.5 μg anti-PA83 IgG fully protected cells against LeTx mediated cell death, while 0.95 μg offered minimal protection (35%) over the LeTx treated control cells (Fig. 2A). Treatment of LeTx exposed cells with anti-PA83 F(ab')_2 _demonstrated equivalent protection at 7.5 μg compared to anti-PA83 IgG (Fig. 2B). At lower doses, there was an observable diminished protection afforded by the anti-PA83 F(ab')_2 _compared to whole IgG. These data confirm that rapidly produced caprine immunotherapeutics, either whole IgG or despeciated F(ab')_2 _fragments, elicit complete protection against LeTx-mediated cytotoxicity *in vitro*.

### *In vivo *protection of mice following LeTx challenge

**Figure 3:**
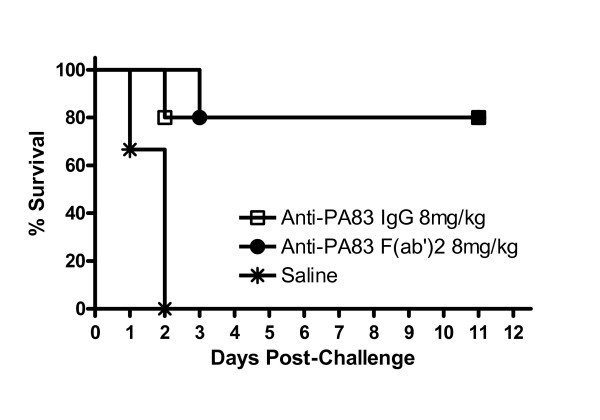
*In vivo *protection against LeTx cytotoxicity. Percent survival of female Balb/c mice treated with 100 μg LeTx by i.p. injection followed 5 minutes later with 8 mg/kg anti-PA83 IgG or F(ab')_2 _antibodies in 200 μl (5 per group). Control mice (Saline, 3 in group) received 100 μg LeTx followed by 200 μl Saline. All mice were observed twice daily for signs of illness or distress and all surviving mice were euthanized at day 11 post-challenge. P < 0.03 by the logrank test.

Efficacy for the anti-PA83 IgG and F(ab')_2 _immunotherapeutics was established in an intraperitoneal LeTx-challenge mouse model (Fig. 3). The LeTx -challenge mouse model simulates a post-exposure, symptomatic patient. Mice were first injected with 2LD_100 _(200 μg LeTx) of recombinant LeTx on the left side of the abdomen. This dose of LeTx has been confirmed to be fatal to 100% of mice within 48 h post challenge (data not shown). After five minutes, mice were injected with approximately 8 mg/kg anti-PA83 IgG or F(ab')_2 _immunotherapeutics on the right side of the abdomen. Control mice received 200 μl of PBS instead of IgG or F(ab')_2_. Control mice succumbed to LeTx by day 2 while IgG and F(ab')_2 _treated groups showed 80% and 100% survival, respectively. F(ab')_2_-treated group survival rates declined to 80% on day 3 and remained there throughout the 11 d study. The IgG-treated group also showed 80% protection for the remainder of the study. The ability for the goat derived passive immunotherapeutic to protect against an *in vivo *LeTx challenge suggests its potential for use as a therapeutic intervention in humans. Since this model simulates a symptomatic patient, we speculated that the anti-PA83 immunotherapeutics could be used efficaciously post-exposure to prevent mortality.

### Passive protection of mice 24 hours post-infection with Ames spores

**Figure 4:**
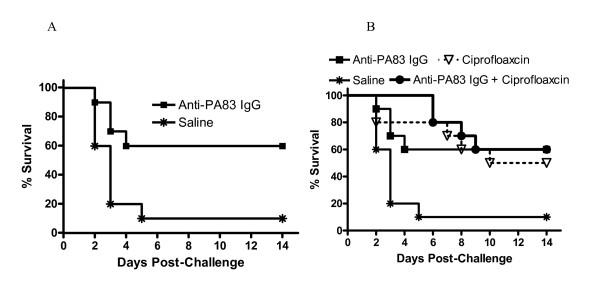
*In vivo *protection against intranasal virulent anthrax challenge. Percent survival of female Swiss Webster mice, 10 per group, infected with 5 LD_50 _*B. anthracis *Ames spores by intranasal inoculation. Control mice were treated with saline 1 h post spore challenge via intraperitoneal injection. All mice were monitored twice dailyfor signs of illness or death. **A**. Mice were treated with 32 mg/kg anti-PA83 IgG 24 h post spore challenge via intraperitoneal injection. P = 0.0161 by thelogrank test. **B**. Mice were treated with Ciprofloxacin alone or in combination with anti-PA83 IgG at 32 mg/kg (24 h post spore challenge). Ciprofloxacin was administered twice daily at 0.9 mg/day via intraperitonealinjection for the first six days post spore challenge. Statistical significance using the logrank test as follows: Anti-PA83 IgG P = 0.0161, Anti-PA83 IgG + Ciprofloaxcin P = 0.0007 and Ciprofloaxcin P = 0.0156.

To evaluate post-exposure efficacy of the anti-PA83 IgG, a mouse model of inhalational anthrax was used. Female Swiss Webster mice were challenged with virulent *B. anthracis *spores via an intranasal infection route. Mice received 5 LD_50 _*B. anthracis *Ames spores in 20 μl instillations into each nares. Control mice received saline at 1 h post-challenge. Twenty-four hours post-challenge, test groups received 32 mg/kg caprine anti-PA83 IgG by intraperitoneal injection. At 4 d post-infection (p.i.), only 20% of control mice survived, while 70% of mice treated with anti-PA83 IgG were still alive (Fig. 4A). By day 6, another 10% of the mice in each group had succumbed to disease and no further mortality was observed through the remaining 14 d study. One test group also received low-dose Ciprofloxacin to examine synergistic effects of post-exposure treatments (Fig. 4B). Mice treated with antibiotics alone exhibited a 50% survival rate out to the end of the study (14 d p.i.). Survival of IgG treated mice dropped to 60% by day 6 p.i. and remained there through the completion of the study. Concomitant administration of Ciprofloxacin (twice daily on days 1–6) and anti-PA83 IgG (single bolus at 24 h p.i.) completely protected mice for 6 days (Fig. 4B) while Ciprofloxacin was administered. When Ciprofloxacin treatment was stopped, survival decreased to levels comparable to anti-PA83 IgG treatment alone. These results confirm the potential for passive transfer of immunity up to 24 hours post exposure to *B. anthracis *spores and suggest parallel treatment with antibiotics can significantly enhance survival.

Many groups have shown the efficacy of polyclonal, animal-derived sera for use as a passive immunotherapeutic against anthrax infections, however these groups have relied on smaller animal models (e.g., mice, rabbits, guinea pigs) to generate the antisera [3,4,26,27]. Smaller animals are typically terminally bled in order to produce larger volumes of serum. Yields from a terminal bleed typically range from 0.5 ml for mice up to 200 ml for terminally bled rabbits. The large number of animals required to produce the therapeutic quantities needed for a useful medical countermeasure stockpile (e.g., the SNS) makes these animal models prohibitively expensive. Caprine plasmapheresis does not require the animals to be euthanized/terminally bled in order to generate large volumes of antisera. Additionally, the goats can be plasmapheresed up to four times per year for several years making for a nearly endless source of antisera. Plasmapheresis of three goats generated liters of anti-PA83 serum within a very short time frame. Additionally, the goats used to produce this material are part of a certified pathogen-free herd and the antisera produced are of GMP grade. Comparably produced IgG against HIV has been previously approved for clinical trials in humans [19-21].

The previously approved AVA anthrax vaccine required a series of six immunizations followed by annual boosts. The use of a novel non-toxic MDP adjuvant enabled the generation of extremely high-titer antiserum following only two immunizations although for the current study, IgG was isolated from goats immunized four times. With further optimization of the immunization regiment, we may be able to generate an efficacious immunotherapeutic with fewer immunizations, thus shortening the production time and cost. It should also be emphasized that the data presented here used non-affinity-purified IgG or F(ab')_2_. Studies are underway to evaluate the efficacy of the affinity purified materials, which may significantly reduce the amount of material required to offer significant protection in both animals and humans.

F(ab')_2 _antibodies have been used for the treatment of rattlesnake bites [28,29], bee stings [30] and evaluated for their potential to treat several infectious diseases including respiratory syncitial virus (RSV) [31]. Many monoclonal antibodies (MAbs) have been generated that are specific for the anthrax protective antigen. The majority of these MAbs do not demonstrate significant protection post-exposure and appear to require a blend of several MAbs in order to reduce the mortality associated with anthrax infections [32,33]. A recent study using a monoclonal antibody against the anthrax protective antigen demonstrated a requirement for the Fc portion of the antibody in order to retain neutralizing capabilities [25]. Our polyclonal immunotherapeutic retained similar neutralizing levels both *in vitro *and *in vivo *after removal of the Fc region by pepsin digestion. These findings are consistent with data from other polyclonal antiserum, which indicate most F(ab')_2 _retain comparable neutralizing and protective abilities to full length IgG [26,29,30,34]. The utility of F(ab')_2 _antisera derived from goats will reduce the potential for side-effects associated with patients who have a pre-existing sensitivity to goat proteins. In addition, patients requiring multiple treatments with an animal derived therapeutic may also be at increased risk of developing allergic hypersensitivity, so the use of F(ab')_2 _antibody fragments will decrease this risk and increase the overall safety of this immunotherapeutic for multiple uses within a large population.

## Conclusion

This work has shown that pharmaceutical-grade goat polyclonal immunotherapeutics specific for the anthrax protective antigen can be rapidly produced in large quantities. Three goats immunized four times over a 56 day period produced liters of GMP grade, high titer antisera that was capable of neutralizing anthrax lethal toxin both *in vitro *and *in vivo*. More importantly the passive transfer of the goat-derived antibodies 24 h post-exposure to virulent anthrax spores provided mice with a substantial survival advantage over untreated mice. A synergistic effect was seen with concomitant antibiotic treatment although levels of protection returned to the levels observed with IgG treatment alone once antibiotic therapy was discontinued. This indicates that a combined treatment approach for patients presenting with clinical signs of anthrax infection could overall increase in survival rates associated with symptomatic disease. Additionally, this immunotherapeutic can be easily produced in quantities large enough to fulfill the requirements for a national medical countermeasures stockpile. The non-toxic MDP adjuvant developed is easily produced; amenable to covalent attachment of antigens, and importantly, renders toxins and pathogens inactive once coupled to the molecule. The use of this novel adjuvant should improve vaccine development and quality control in addition to eliciting significantly higher immune responses than standard adjuvants.

## Competing interests

Portions of these studies were funded by Virionyx Corporation Ltd who hold patent rights to the non-toxic MDP adjuvant.

## Authors' contributions

CDK performed all *in vitro *and *in vivo B. anthracis *lethal toxin assays and was primary author on this manuscript. CO and FBG provided NT-MDP, immunized goats, purified IgG fractions, isolated F(ab')_2 _fractions, and contributed to writing this manuscript. JWP and LES performed *B. anthracis *infectious murine *in vivo *assays. NMC provided study designs and contributed to writing this manuscript.
